# The Effects of Metal Complexes of Nano-Graphene Oxide to Thermal Decomposition of FOX-7

**DOI:** 10.3390/nano10010144

**Published:** 2020-01-13

**Authors:** Chongmin Zhang, Xiaolong Fu, Xuexue Zhang, Jizhen Li, Xuezhong Fan, Guofang Zhang

**Affiliations:** 1 Xi’an Modern Chemistry Research Institute, Xi’an 710065, China; iceand010@163.com (C.Z.); jizhenli@126.com (J.L.);; 2 Science and Technology on Combustion, Internal Flow and Thermostructure Laboratory, Northwestern Polytechnical University, Xi’an 710072, China; xuexuezhang@mail.nwpu.edu.cn; 3 Key Laboratory of Applied Surface and Colloid Chemistry, MOE/School of Chemistry and Chemical Engineering, Shaanxi Normal University, Xi’an 710062, China; gfzhang@snnu.edu.cn

**Keywords:** nano-graphene oxide, energetic materials, thermal decomposition, physical chemistry

## Abstract

1,1-diamino-2,2-dinitroethene (FOX-7) an insensitive high-energetic compound, has rarely been reported previously with respect to combustion performance. In order to quickly apply it to propellants, the thermal decomposition behaviors of FOX-7 should be optimized. Metal complexes of nano-graphene oxide have an obvious effect on the thermal decomposition of energy materials. In this paper, the metal (Cu^2+^ and Fe^3+^) complexes of nano-graphene oxide (nGO) have been synthesized using nGO as a ligand and metal ions as a coordination center. They were mixed with FOX-7 as additives to form energetic composites, and the chemical bond distributions and thermal decomposition processes of these composites have been investigated. The results show that Cu^2+^ and Fe^3+^ are uniformly distributed on the surface of the nGO. The thermal decomposition processes of nGO-metal-FOX-7 composites have been fully characterized by Differential scanning calorimeter (DSC) and Thermal gravimetric analyzer-Differential scanning calorimeter-Infrared spectroscopy-Mass spectrum (TG-DSC-IR-MS) coupling technology. The results show that the Fe^3+^ based complex affect the decomposition of FOX-7 than the Cu^2+^ based one with the initial decomposition temperature of 230 °C and the apparent heat release of 3535.6 J·g^−1^. Moreover, the addition of nGO-metal complexes could promote the formation of nitrogen during the decomposition of FOX-7, indicating a more complete decomposition.

## 1. Introduction

Solid propellants are mainly used in weapons, such as rockets and missiles that contain propulsion systems [1,2,3,4,5,6]. Energetic materials were used in propellants, explosives, and pyrotechnics, which contain different ingredients depending on the application [7,8,9]. The requirements of solid propellants for energetic materials mainly include large gas production, fast decomposition, high energy, and low sensitivity [10,11,12]. The content of additives is generally lower compared to other ingredients, but its influence on the performance of solid propellants is indeed enormous [13,14,15,16,17]. In particular, improvement in the combustion properties of metal additives on solid propellants is significant, such as Al, CuO and Fe_2_O_3_ [18,19,20,21,22]. However, when metal powders or metal oxides are added, they tend to agglomerate when the solid propellant is burned [23,24,25], which affects the combustion properties of propellants.

Graphene has been used in the field of energetic materials and good achievements have been obtained about catalytic thermal decomposition and reducing sensitivity of propellants [26,27,28,29,30,31,32]. Graphene can significantly reduce the two decomposition peak temperature of ammonium perchlorate (AP), and even allow the low temperature decomposition peak disappear [33,34]. Moreover, it accelerates electron transfer in AP decomposition and effectively increases the reaction rate [35,36]. When graphene oxide is loaded with nano-metal tungstate, it can promote the reduction of decomposition peak temperature and activation energy and the increase of exotherm of RDX [37]. The addition of GO and neutral polymeric bonding agent (NPBA)can improve the mechanical properties of 1,3,5-triamino-2,4,6-trinitrobenzene (TATB)-based polymer bonded explosives (PBXs) [38]. Therefore, both safety and apparent heat release rate of solid propellants could be increased after the addition of graphene-based additives. Meanwhile, since graphene was a two-dimensional (2D), material with high theoretical surface area and excellent electronic and thermal conductivity [39], it could be a good metal ion carrier [36,40,41].

FOX-7 is an insensitive high density energetic material with a theoretical density of 1.885 g·cm^−3^, with detonation performance close to that of hexahydro-1,3,5-trinitro-1,3,5-triazine (RDX) [42,43,44]. Due to its intramolecular and intermolecular hydrogen bonding, FOX-7 is compatible with a variety of materials, such as RDX and 1,3,5,7-tetranitro-1,3,5,7-tetrazacyclooctane (HMX) [45]. But the sensitivity of FOX-7 is lower than that of RDX and HMX [46,47,48]. Based on these properties, FOX-7 could be used as a substitute for RDX in solid propellants [49,50]. However, FOX-7 is a new material that has rarely been reported in relation to its combustion performance. Therefore, in order to quickly apply FOX-7 to the propellants, it is necessary and meaningful to optimize the thermal decomposition behaviors of FOX-7 in the presence of additives.

Based on the above points, the nano-graphene oxide (nGO) could be a ligand coordinated with metal ions to solve the agglomeration problem of the metal additives, for the thermal decomposition research of FOX-7.

In this paper, nGO is used as a ligand for metal ions (Cu^2+^ and Fe^3+^) in order to obtain the metal complexes of nGO as additives for thermal decomposition of FOX-7 and its thermodynamic properties were characterized. At the same time, the gaseous products produced by their decomposition are also summarized. This study helps to improve the combustion performance and broaden the application prospects in solid propellants of FOX-7.

## 2. Experimental

### 2.1. Materials and Methods

Graphene oxide (diameter: 1–5 µm, thickness: 0.8–1.2 nm, single layer ratio: >99%) was purchased from Nanjing JCNANO Tech Co., Ltd (Nanjing, China). Iron (III) nitrate nonahydrate (>98.5 wt % purity) and cupric nitrate (>99.5 wt % purity) were commercially available from Tianjin Fuchen Chemical Reagents Factory (Tianjin, China). FOX-7 is synthesized by Xi’an Modern Chemistry Research Institute (>99.5 wt % purity). Nano iron oxide (particle size: 50 nm, >99.9 wt % purity) and nano-copper oxide (particle size: 40 nm, >99.9 wt % purity) were acquired from the Shanghai Chaoweinano Tech Co., Ltd (Shanghai, China).

### 2.2. Preparation of nGO-Metal Complexes

The first procedure is the dispersion of nano-graphene oxide. Nano-graphene oxide was weighed 100 mg and added to 100 mL of water. The solution was magnetically stirred for 30 min at room temperature. Thereafter, the aqueous nano-graphene oxide solution was ultrasonicated for 1 h until it is uniformly dispersed in water. A total of two sets of nGO aqueous solutions were prepared. We weighed 600 mg each of iron (III) nitrate nono-hydrate and cupric nitrate and added them separately to the previously prepared dispersed aqueous solution of nGO. In order to bring the nGO and metal ions into full contact, both solutions were sonicated for 1 h. Then, the two solutions were heated to 70 °C with magnetic stirring for 3 h. After the solution reaction was completed, the precipitate was collected by vacuum suction filtration and washed twice with water. The precipitate was vacuum-dried at −20 °C. The prepared nGO-metal complexes were tested by infrared, Raman, XPS, and scanning electron microscopy, in order to characterize their structure and chemical bonds.

### 2.3. Preparation of nGO-Metal-FOX-7 Composites

The prepared nGO-Cu and nGO-Fe complexes were mixed with FOX-7 respectively and ground using a semi-solvent method with ethanol as solvent to form nGO-Cu-FOX-7 and nGO-Fe-FOX-7. Among them, the mass fraction of nGO-Cu and nGO-Fe is 20%. nGO, nano-Fe_2_O_3_, and nano-CuO were mixed with FOX-7 to form nGO-FOX-7, Fe_2_O_3_-FOX-7 and CuO-FOX-7 using the above method as control groups. The prepared mixtures were characterized by Differential scanning calorimeter (DSC) and Thermal gravimetric analyzer-Differential scanning calorimeter-Infrared spectroscopy-Mass spectrum (TG-DSC-IR-MS) coupling technology test, respectively, in order to study their thermal decomposition process and products.

## 3. Results and Discussion

### 3.1. Chemical Bonding and Morphology

**Figure 1:**
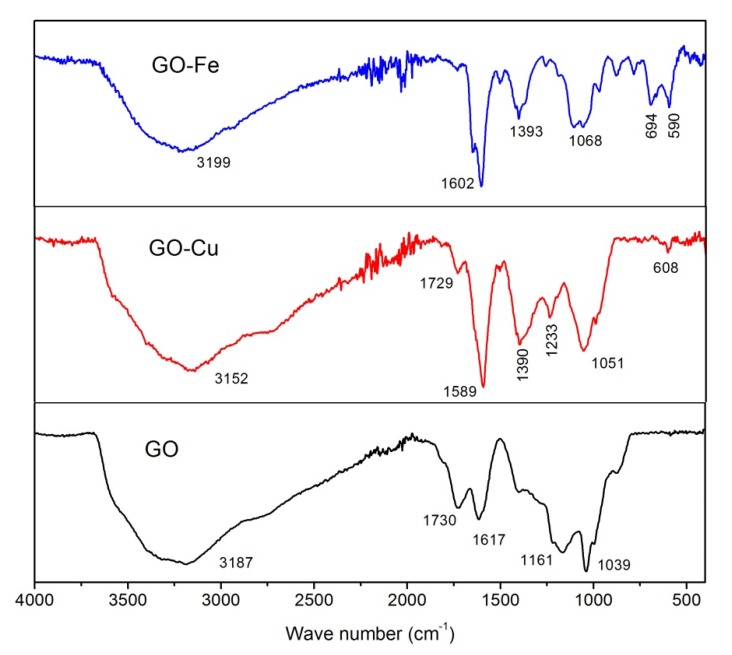
The fourier-transform infrared spectroscopy (FTIR) spectra of nGO, nGO-Cu and nGO-Fe.

The FTIR analysis was performed to confirm the chemical bonding in nGO and its complexes. As shown in Figure 1, Table S1, and Figure S6 (supporting information), the existence of some chemical bonds in nGO, nGO–Cu, and nGO-Fe are confirmed. The peaks in 500–750 cm^−1^ are attributed to stretching of metal-O bond. In Figure 1, the peak at around 600 cm^−1^ demonstrated the existence of metal ions. This shows that nGO is well-coordinated with the metal ions. The peak at around 1600 cm^−1^ belongs to the carboxylic and/or carbonyl moiety functional groups or the bending vibration of O–H. The stretching of C=O contributes to the peak at 1730 cm^−1^. It is interesting to note that after coordination, the peak of C=O stretching in nGO almost disappears. This may be accounted for by the loss of protons by two carboxylic groups involved in the coordination of a single metal center. The peak at 1150–1400 cm^−1^ corresponds to the stretching of C–O in carboxyl group and C–O–C stretching vibration for epoxy group. After the coordination, the number of peaks increases and the peak region becomes complicated at 1150–1400 cm^−1^. This could be attributed to the effect of coordination of metal ions with oxygen atoms. The peaks at 1000–1110 cm^−1^ are the result of stretching of C–OH bond. The broad peak at 3000–3600 cm^−1^ is due to the H_2_O absorbed by nGO and the stretching of O–H. At 2850–2930 cm^−1^ the absorption corresponds to inversely symmetrical and symmetrical stretching vibration of CH_2_ [51].

**Figure 2:**
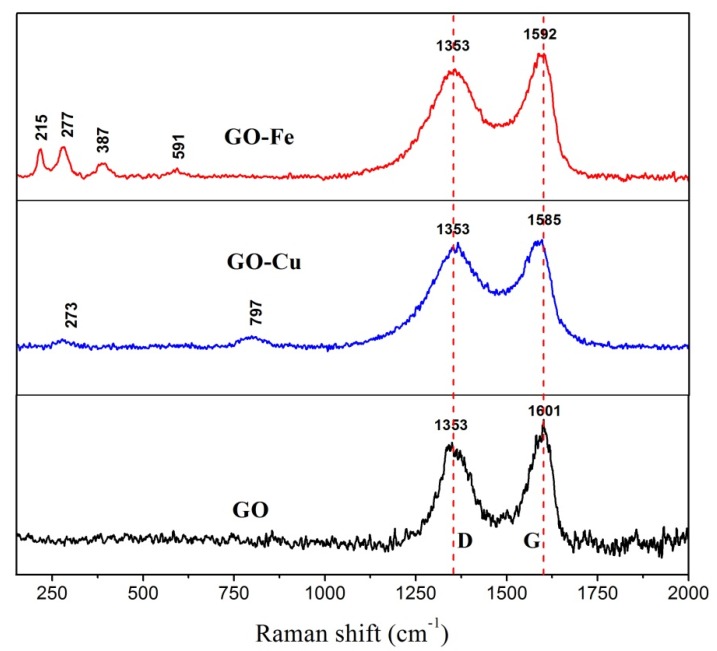
Raman spectra of nGO, nGO-Cu, and nGO-Fe.

The obtained nGO, nGO-Cu and nGO-Fe were further investigated by Raman spectroscopy. As shown in Figure 2, the Raman spectrum of GO has two peaks at 1353 and 1601 cm^−1^, which are D- and G-bands, as reported in the reference [52]. The formation of the G-band could be attributed to the in-phase vibration of graphite lattice, and the D-band is related to the disorder band caused by the graphite edges. After coordination, G band of GO is shifted towards lower wave number. This could be caused by the recovery of hexagonal network of carbon atoms with defects [53]. The Raman spectra of the nGO-Cu and nGO-Fe also shows D- and G-band with comparable I_D_/I_G_ intensity ratios to that of GO, indicating that the skeleton structure of nGO is preserved (Table S2). It is particularly note that various Raman bands caused by iron oxide such as Fe_2_O_3_ (406, 657, and 670 cm^−1^), Fe_3_O_4_ (603 and 670 cm^−1^), and FeOOH (218, 285, 289 and 397 cm^−1^) [54] appeared in the Raman spectra of 100–600 cm^−1^ in Figure 2 after the nano-graphene oxide complexed with Fe^3+^. Simultaneously, the Raman spectrum of nano-graphene oxide coordinated with Cu^2+^ exhibits several peaks at the range of 200–800 cm^−1^. These Raman peaks are corresponding to some different cooper oxides, such as CuO (299, 342, 500, 634 cm^−1^), Cu(OH)_2_ (450–470, 540–580 cm^−1^), and Cu_2_O (214, 644 cm^−1^) [55,56]. Therefore, from the results of the Raman spectrum, the nano-graphene oxide is successfully coordination with Cu^2+^ and Fe^3+^, which affects the D-band and the G-band of nano-graphene oxide.

**Figure 3:**
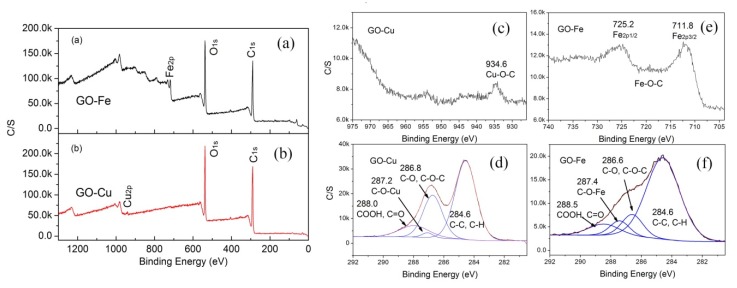
X-ray photoelectron spectroscopy (XPS) binding energy spectra of (**a**) nGO-Fe, (**b**) nGO-Cu; (**c**) the Cu 2p spectra of nGO-Cu, without peak fitting; (**e**) the Fe 2p spectra of nGO-Fe, without peak fitting; the fitted C 1s peak curves for (**d**) nGO-Cu, and (**f**) nGO-Fe.

In order to better study the distribution of chemical bonds in nGO, nGO-Cu, and nGO-Fe, X-ray photoelectron spectroscopy (XPS) was performed. In nano-graphene oxide, the C 1s peak includes C–C bonds (284.6 eV), C–O bonds (286.8 eV), and C=O bonds (288.6 eV), as shown in Figure S1. Figure 3 shows the XPS spectrum of nGO-Cu and nGO-Fe. The peak was shown in Cu 2p^2/3^ curves at 934.6 eV (Figure 3c). From the results of EDS, it is known that the content of Cu^2+^ on nGO is less than that of oxygen atoms. Therefore, in the C 1s curve of nGO-Cu, the peak at 286.8 eV should mainly belong to C–O rather than C–O–Cu (Figure 3e). Figure 3d shows two peaks of Fe 2p^1/2^ and Fe 2p^3/2^. In Figure 3f, the peak at 286.6 eV belongs to C–O in nGO and the peak at 287.4 eV should belong to C–O–Fe.

**Figure 4:**
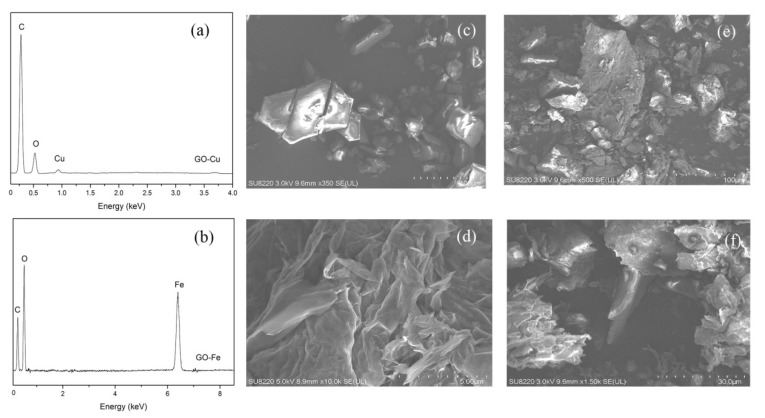
EDS spectra surface elemental analysis of (**a**) nGO-Cu and (**b**) nGO-Fe; SEM images of (**c**) FOX-7, (**d**) nGO, (**e**) nGO-Fe-FOX-7, (**f**) nGO-Cu-FOX-7.

Figure 4 shows the SEM images and Ehlers-Danlos syndrome (EDS) curves of nGO, nGO-Cu, and nGO-Fe. It can be seen from the EDS results that the content of Cu^2+^ in nGO is lower than that of Fe^3+^, indicating that nGO is easier to form complex with Fe^3+^. It could be known from the SEM images that FOX-7 is a large crystal grain with a regular surface, and nGO is hierarchical. In Figure 4e,f, the mixing of FOX-7 and nGO could be confirmed, which should be on the micron level.

Multi-elemental EDS mapping images of C, O, Cu, and Fe are shown in Figures S4 and S5. The distribution of C, O, and metal ions can be seen from the images, and the results show that the metal ions are uniformly distributed on the nano-graphene oxide.

### 3.2. The Thermal Decomposition Behavior

**Figure 5:**
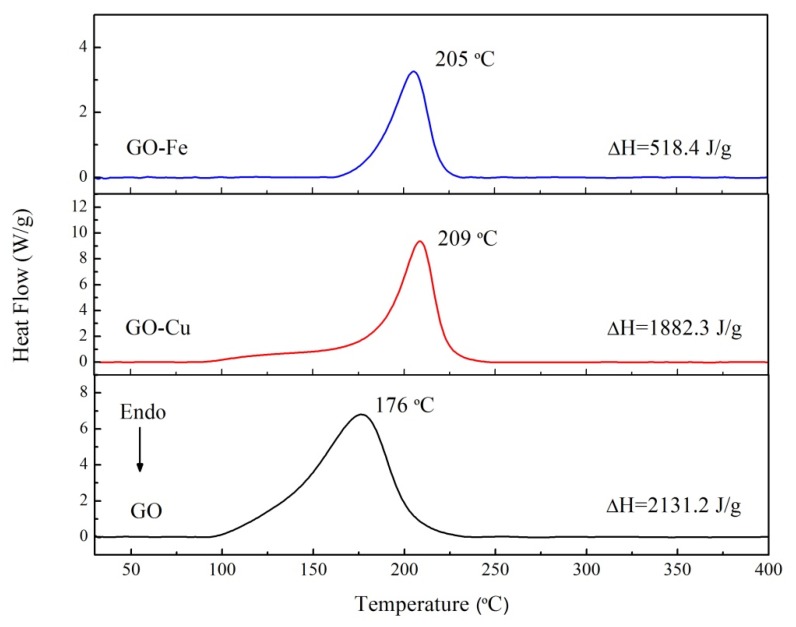
DSC curves of nGO, nGO-Cu, and nGO-Fe with heating rate of 10 °C/min.

Figure 5 shows the DSC results of nGO, nGO-Cu, and nGO-Fe. The decomposition temperature of nGO is 176 °C, and that of nGO-Cu and nGO-Fe are 209 °C, 205 °C, respectively. This indicates that the thermal stability of nGO is improved after the Cu^2+^ and the Fe^3+^ are respectively coordinated with the oxygen atom on nGO. This is because the dissociation energy of the O-Cu (Fe) bond is higher than that of O-H bond in nGO. It could be inferred from the magnitude of the heat release that the coordination between nGO and Fe^3+^ is tighter. Because nGO is coordinated with metal ions, that causes a decrease in heat release, especially in Fe^3+^. This shows that the amount of Fe^3+^, that is coordinated with nGO, is more than that of Cu^2+^, which is consistent with the EDS result. Since copper nitrate and iron(III) nitrate nonahydrate are added in the same amount in the reaction, it is considered that Fe^3+^ are more suitable to be coordinated with nGO than Cu^2+^.

**Figure 6:**
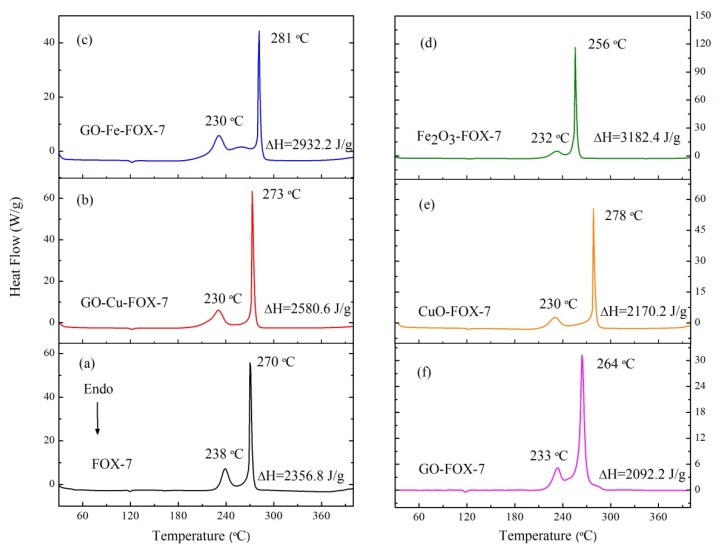
DSC curves of (**a**) FOX-7, (**b**) nGO-Cu-FOX-7, (**c**) nGO-Fe-FOX-7, (**d**) Fe_2_O_3_-FOX-7, (**e**) CuO-FOX-7 and (**f**) nGO-FOX-7 with heating rate of 10 °C/min.

There are two consecutive exothermic stages in the decomposition process of FOX-7 (Figure 6a), which are low temperature exotherm (220–250 °C), and high temperature exotherm (260–280 °C), respectively. In this paper, we mainly focus on the low temperature exothermic stage of FOX-7, which is also the initial decomposition peak as it is more representative of the initial decomposition process of FOX-7 than the second exothermic peak.

The initial decomposition peak temperature of FOX-7 is 238 °C. After coordination with metal ions, as can be seen from Figure 6b,c, the decomposition temperature of nGO-Cu-FOX-7 and nGO-Fe-FOX-7 composites are both 230 °C, which are reduced by 8 °C compared to FOX-7. In Figure 6c, there is a small peak at about 250 °C. By comparing the ion current data of *m/z* = 30 (Figure S7), it can be seen that the ion current of nGO-Fe-FOX-7 is the highest at about 250 °C compared to others. This shows that, at this temperature, NO will be generated in large quantities. Therefore, the small peak at about 250 °C could be attributed to the generation of NO. In comparison, the thermal stability of Fe_2_O_3_-FOX-7, CuO-FOX-7 and nGO-FOX-7 composites are also been investigated. The initial decomposition temperature of Fe_2_O_3_-FOX-7, CuO-FOX-7 and nGO-FOX-7 are all reduced compared to FOX-7. This shows that, not only metal ions, but also nGO itself can decrease the initial decomposition temperature of FOX-7, which indicates that nGO also has a certain effect on FOX-7. In Figure 6d, the distance between the two decomposition peak temperatures is closer, which means that it will release heat more quickly. At the same time, combined with the results of heat release, Fe^3+^ can indeed promote the exothermic heat of FOX-7. Therefore, Fe^3+^ may have a stronger effect on the decomposition process of FOX-7.

The apparent decomposition heat release of nGO, FOX-7 and their complexes (the sum of the two peak areas) are listed in Table 1 by integrating the heat flow values of the DSC curve of the samples. Since the additives are contained in the mixture, the apparent decomposition heat release is standardized. As can be seen from Table 1, the standard apparent decomposition heat release of FOX-7 is 2356.8 J·g^−1^. In the series of FOX-7 mixtures, only the standardized value of nGO-FOX-7 is lower than that of FOX-7. This shows that the thermal decomposition process of FOX-7 is more affected by the addition of metal ions. At the same time, the promotion of the exothermic process of FOX-7 by Fe^3+^ is very obvious. In the nGO-Fe-FOX-7 and Fe_2_O_3_-FOX-7 samples, the apparent decomposition heat release of FOX-7 is as high as 3535.6 and 3978.0 J·g^−1^. From the heat release results of CuO-FOX-7, it is shown that the effectof Cu^2+^ to FOX-7 is not significant, compared to Fe^3+^. At the same time, the results of EDS and Raman show that nGO has a stronger coordination effect with Fe^3+^. This indicates that Fe^3+^ are more suitable for the decomposition system of FOX-7 than Cu^2+^.

### 3.3. The Thermal Decomposition Process

**Figure 7:**
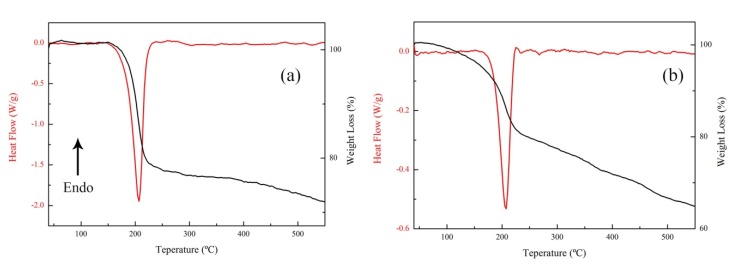
TG-DSC curves of (**a**) nGO-Cu and (**b**) nGO-Fe.

**Figure 8:**
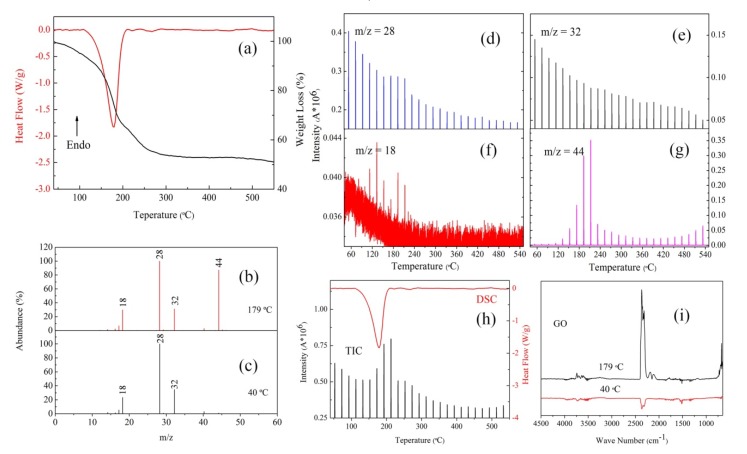
(**a**) DSC and TG curves of nGO with heating rate of 10 °C/min; the MS spectra of nGO at (**b**) 179 °C and (**c**) 40 °C; the ion current spectra of nGO in the decomposition with (**d**) *m/z* = 28, (**e**) *m/z* = 32, (**f**) *m/z* = 18, (**g**) *m/z* = 44, (**h**) total ion current; (**i**) IR spectra of nGO at 179 °C and 40 °C.

In order to further study the thermal decomposition process and decomposition products of FOX-7 and nGO-metal-FOX-7 composites, the samples, such as nGO, nGO-Cu, nGO-Fe, FOX-7, nGO-Cu-FOX-7, and nGO-Fe-FOX-7 were investigated by using on-line TG-DSC-IR-MS techniques. From the results of TG (Figure 7 and Figure 8a), the last remaining residue of nGO-Cu and nGO-Fe is higher than that of nGO. This proves that after coordination with metal ions, nGO becomes more difficult to decompose. This can explain that in the DSC results, the initial decomposition temperature of nGO-metal complexes is 30 °C higher than that of nGO. From the TG results of FOX-7, the residual mass of FOX-7 was almost zero, indicating that FOX-7 was completely decomposed into gaseous products in the test.

According to the MS result of nGO (Figure 8b,c), the decomposition products can be investigated. The initial decomposition process of nGO mainly undergoes decomposition of oxygen-containing functional groups, including detachment of hydroxyl groups, decomposition of carboxyl groups into hydroxyl radicals, and carbon monoxide, and the radical reaction of hydroxyl radicals with hydroxyl groups and carboxyl groups on nGO [57]. Therefore, its main decomposition products include water, carbon monoxide, carbon dioxide, etc. The carbon ring of nGO produces almost no gas products during the decomposition process, but rather rearranges or forms defects, which is consistent with the TG results.

At 40 °C, there are mainly three peaks, which are *m*/*z* = 18, 28, and 32. These three peaks represent H_2_O, N_2_, and O_2_, which are components of air. At 179 °C, a peak of *m*/*z* = 44 appeared. According to the composition of graphene, it can be inferred that this is carbon dioxide. Compared to 40 °C, the ratio of peaks *m*/*z* = 18 to *m/z* = 32 increases, indicating that nGO has water formation at the time of decomposition. According to reference [57], the product represented by *m/z* = 28 may contain carbon monoxide at 179 °C.

From the results of IR, it is known that the main gas products produced by the decomposition of nGO are H_2_O, CO_2_ and CO. In Figure 8i, the peak at 1200–1600 cm^−1^ and 3200–4000 cm^−1^ are caused by water vapor. The peak at 2250–2400 cm^−1^ is caused by CO_2_ and the peak at 2050–2250 cm^−1^ corresponds to CO, which proves that the *m/z* = 28 is also belongs to CO in Figure 8b,c.

The total ion current results were shown in Figure 8h. When the temperature reaches 180 °C, there are a large number of ion current generations. However, the total ion current is not zero in the beginning due to the presence of air. It is known that the ionic intensity of water is small from Figure 8f, while that of carbon dioxide is the highest. At 220 °C, the ion current of carbon dioxide accounts for almost half of the total ion current, which means the main gas product of nGO is carbon dioxide during thermal decomposition. The ion current of *m/z* = 28 and 32 changes little at 150–250 °C due to effect of air. However, due to the formation of carbon monoxide, the curve of *m/z* = 28 has a certain change at 150–210 °C.

**Scheme 1:**
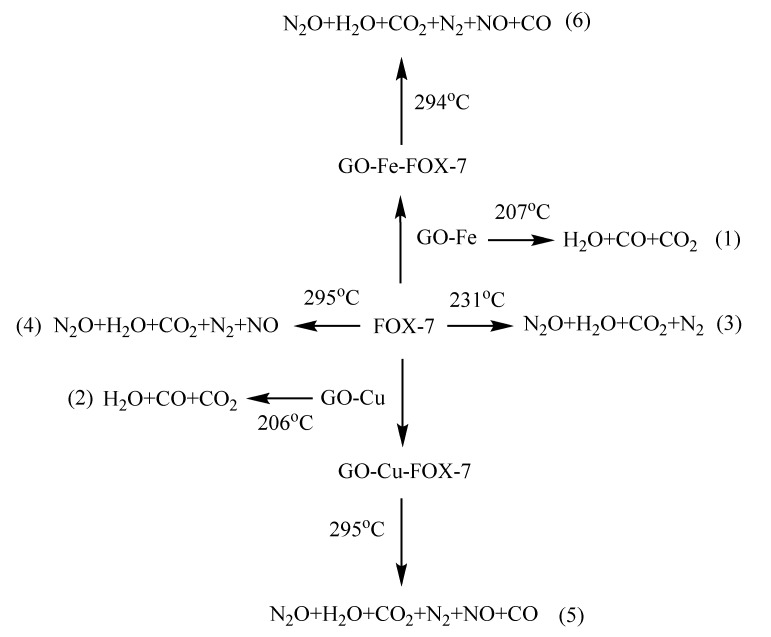
The decomposition process of FOX-7 and its derivatives.

The decomposition process of nGO-Cu and nGO-Fe are almost same with that of nGO (Figures S2 and S3). Since the metal ions act as a catalyst during the decomposition process of nano-graphene oxide, the kind of decomposition products are not changed. It is only due to the effect of the coordination that the decomposition temperature of nGO is delayed by about 30 °C. The decomposition process of nGO-Cu and nGO-Fe were shown in (1) and (2) of Scheme 1.

**Figure 9:**
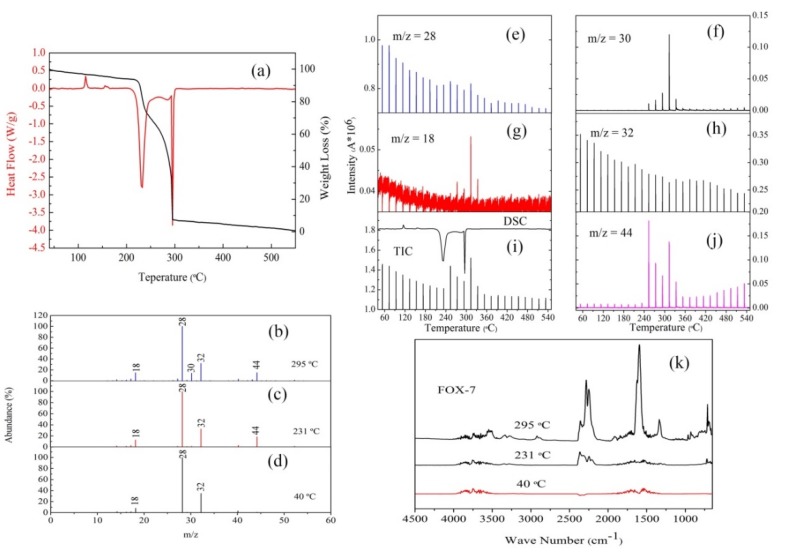
(**a**) DSC and TG curves of FOX-7 with heating rate of 10 °C/min; the MS spectra of FOX-7 at (**b**) 295 °C, (**c**) 231 °C and (**d**) 40 °C; the ion current spectra of FOX-7 in the decomposition with (**e**) *m/z* = 28, (**f**) *m/z* = 30, (**g**) *m/z* = 18, (**h**) *m/z* = 32, (**i**) total ion current, (**j**) *m/z* = 44; (**k**) IR spectra of FOX-7 at 295 °C, 231 °C and 40 °C.

Figure 9 shows the TG-DSC-IR-MS results of FOX-7. At the first decomposition peak (231 °C), *m/z* = 44 appeared compared to 40 °C. It can be inferred from the structure of FOX-7 that it belongs to N_2_O or CO_2_. At second decomposition peak (295 °C), a new peak with *m/z* = 30, has been added. We speculate that this peak belongs to NO. This indicates that nitric oxide does not occur during the high temperature exotherm. This is because nitric oxide is more stable compared to nitrous oxide.

As can be seen from the results of the MS, only N_2_O or CO_2_ were produced in large quantities at 231 °C compared to 40 °C. The result of IR also supports this result. In Figure 9k, the peak 2200–2500 cm^−1^ are caused by N_2_O or CO_2_. At 295 °C, the peaks at 1600 cm^−1^ and 1300 cm^−1^ belong to NO, which is consistent with the MS results. At high temperatures, the peak intensity increases, indicating that FOX-7 experienced a more severe decomposition.

During the decomposition process, water production is small, which is consistent with the weak peak of the water vapor in the infrared result. Based on the data of *m/z* = 28 only little nitrogen is generated at 250 °C. From the results of *m/z* = 30 and 44, N_2_O and CO_2_ are generated in two violent decompositions, and NO is generated only in the second decomposition process. *m/z* = 32 has almost no change between 240–300 °C, indicating that it is only affected by air and has no relationship with the thermal decomposition process of FOX-7. Based on the TG results, FOX-7 was almost completely decomposed into a gas product. Nitrogen was produced in a small amount in the decomposition, while the production of nitric oxide and nitrous oxide was large, which indicates that the N atoms in FOX-7 mainly form a covalent bond with the O atoms in the decomposition. The decomposition process of FOX-7 was shown in (3) and (4) of Scheme 1.

**Figure 10:**
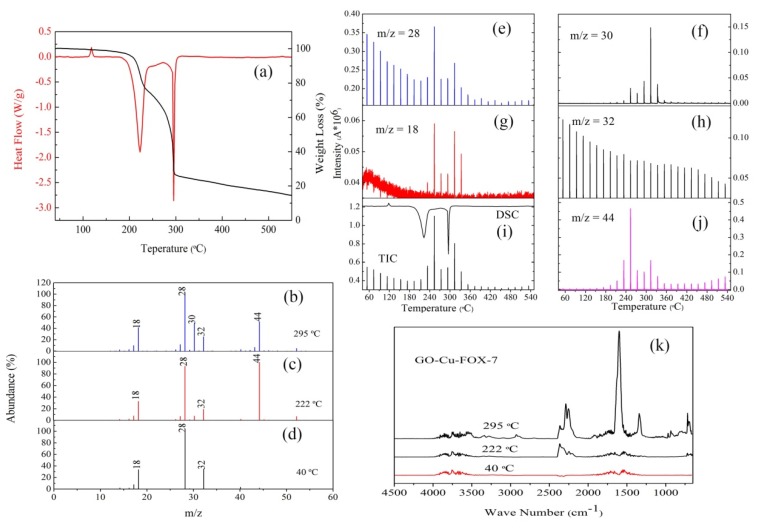
(**a**) DSC and TG curves of nGO-Cu-FOX-7 with heating rate of 10 °C/min; the MS spectra of nGO-Cu-FOX-7 at (**b**) 295 °C, (**c**) 222 °C and (**d**) 40 °C; the ion current spectra of nGO-Cu-FOX-7 in the decomposition with (**e**) *m/z* = 28, (**f**) *m/z* = 30, (**g**) *m/z* = 18, (**h**) *m/z* = 32, (**i**) total ion current, (**j**) *m/z* = 44; (**k**) IR spectra of nGO-Cu-FOX-7 at 295 °C, 222 °C and 40 °C.

The decomposition results of nGO-Cu-FOX-7 were shown in Figure 10. At 222 °C, the ratio of peaks *m/z* = 44 to *m/z* = 28 increases compared to pure FOX-7 in Figure 10c. This indicates that the production of carbon dioxide is increased after mixing with nGO. It can be seen from the ion current data of *m/z* = 44 in Figure 10j that the N_2_O and CO_2_ were generated at 230–330 °C, which indicates that the decomposition temperature of nGO was delayed. Nitrogen and carbon monoxide are produced in large quantities at around 240 °C. At the first decomposition stage, the products are mainly N_2_, CO, CO_2_, and N_2_O, while in the second decomposition stage, the products are mainly N_2_, CO, NO, CO_2_, and N_2_O. The IR results of nGO-Cu-FOX-7 are similar to that of FOX-7, which proves that carbon dioxide is produced in the decomposition of FOX-7 (Figure 10k). Unlike the decomposition process of FOX-7, the peak intensity of *m/z* = 28 is increased (Figure 10e). In this system, *m/z* = 28 may be attributed to carbon monoxide or nitrogen. However, from the results of the infrared, the peak of nGO-Cu-FOX-7 at 2200–2500 cm^−1^ is more similar to that of FOX-7 than nGO, which means that the amount of carbon monoxide in the decomposition product of nGO-Cu-FOX-7 is small. Therefore, the formation of nitrogen in the decomposition of FOX-7 is promoted after the addition of nGO-Cu. The decomposition process of nGO-Cu-FOX-7 was shown in (5) of Scheme 1.

**Figure 11:**
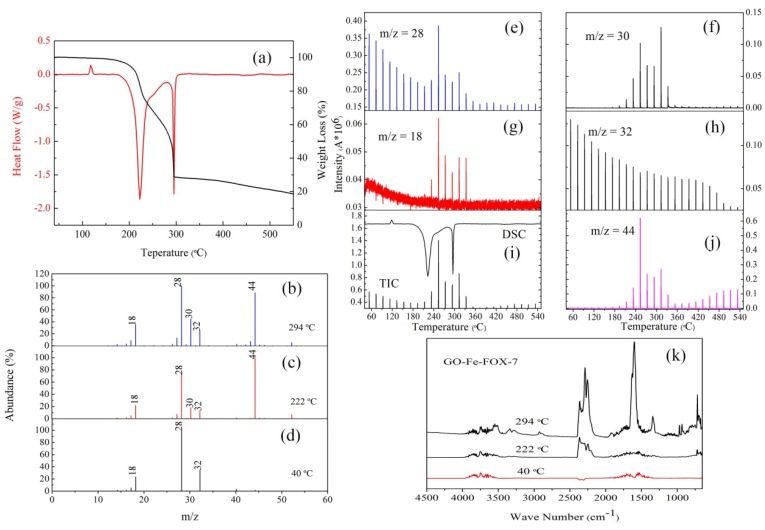
(**a**) DSC and TG curves of nGO-Fe-FOX-7 with heating rate of 10 °C/min; the MS spectra of nGO-Fe-FOX-7 at (**b**) 294 °C, (**c**) 222 °C and (**d**) 40 °C; the ion current spectra of nGO-Fe-FOX-7 in the decomposition with (**e**) *m/z* = 28, (**f**) *m/z* = 30, (**g**) *m/z* = 18, (**h**) *m/z* = 32, (**i**) total ion current, (**j**) *m/z* = 44; (**k**) IR spectra of nGO-Fe-FOX-7 at 294 °C, 222 °C and 40 °C.

The decomposition products of nGO-Fe-FOX-7 are similar to that of nGO-Cu-FOX-7 (Figure 11). However, based on the ion current of *m/z* = 30 it is known that the generation of NO is higher in nGO-Fe-FOX-7. This may be because the coordination effect of Fe^3+^ with nGO is higher. Therefore, the thermal decomposition of FOX-7 proceeds more thoroughly, which are consistent with the DSC result. The decomposition process of nGO-Fe-FOX-7 was shown in (6) of Scheme 1.

## 4. Conclusions

In this work, we constructed metal (Cu^2+^ and Fe^3+^) complexes of nGO and studied their effect on the thermal decomposition process of FOX-7.

(1) nGO is used as a ligand to coordinated with Cu^2+^ and Fe^3+^. The C-O-Cu (Fe) were confirmed to exist in the metal (Cu^2+^ and Fe^3+^) complexes of nGO by IR and Raman spectroscopy and XPS and EDS techniques, indicating nGO successfully coordinated with Cu^2+^ and Fe^3+^.
(2) It can be seen from the SEM images that the nGO-metal complexes is uniformly mixed with FOX-7. After mixing with nGO-metal complexes, the thermal decomposition temperature of FOX-7 is decreased, and the heat release is increased, especially after the addition of Fe^3+^. This indicates that nGO-metal complexes affect obviously on the decomposition process of FOX-7.
(3) After complexing with metal ions, the thermal decomposition temperature of nGO was delayed by 30 °C, but the type and proportion of decomposition products did not change. FOX-7 produces N_2_O, NO and a small amount of H_2_O during the decomposition process. After mixing with nGO-metal complexes, the formation of nitrogen during the decomposition of FOX-7 has been increased, indicating the complete decomposition of that.

## Figures and Tables

**Table 1:** Apparent decomposition heat of nGO, FOX-7 and their complexes.

Sample	Apparent Decomposition Heat/J·g^−1^	
	Test Value	Standardized Value
nGO	2132.2	2132.2
nGO-Cu	1882.3	1882.3
nGO-Fe	518.4	518.4
FOX-7	2356.8	2356.8
nGO-FOX-7	2092.2	2082.4
nGO-Cu-FOX-7	2580.6	2755.2
nGO-Fe-FOX-7	2932.2	3535.6
CuO-FOX-7	2170.2	2712.7
Fe_2_O_3_-FOX-7	3182.4	3978.0

## Supplementary Materials

The following are available online at https://www.mdpi.com/2079-4991/10/1/144/s1, Figure S1: XPS binding energy spectra of (a) nGO and (b) the fitted C 1s peak curves for nGO, Figure S2: The MS spectra of nGO-Cu at (a) 206 °C and (b) 40 °C; (c) IR spectra of nGO-Cu at 206 °C and 40 °C, the ion current spectra of nGO-Cu in the decomposition with (d) *m/z* = 28, (e) *m/z* = 32, (f) *m/z* = 18, (g) *m/z* = 44, (h) total ion current, Figure S3: The MS spectra of nGO-Fe at (a) 207℃ and (b) 40℃; (c) IR spectra of nGO-Fe at 207℃ and 40℃; the ion current spectra of nGO-Fe in the decomposition with (d) *m/z* = 28, (e) *m/z* = 32, (f) *m/z* = 18, (g) *m/z* = 44, (h) total ion current, Figure S4: Mutil-elemental EDS mapping images of nGO-Cu: (a) SEM image, the distribution of (b) C, (c) O, (d) Cu atoms, Figure S5: Mutil-elemental EDS mapping images of nGO-Fe: (a) SEM image, the distribution of (b) C, (c) O, (d) Fe atoms, Figure S6: The comparison of GO-Cu and GO-Fe in FTIR, Figure S7: The ion current spectra of FOX-7, nGO-Fe-FOX-7 and nGO-Cu-FOX-7 in the decomposition with *m/z* = 30, Table S1: FTIR peaks and peak assignments for nGO, nGO-Cu and nGO-Fe.
