# Supplementary material for: The Effects of Metal Complexes of Nano-Graphene Oxide to Thermal Decomposition of FOX-7
Source: Nanomaterials (Basel). 2020 Jan 13;10(1):144. doi: 10.3390/nano10010144 (PMC7023237; doi:10.3390/nano10010144)
Supplement: Supplementary file 1 [file nanomaterials-10-00144-s001.pdf]

The Effects of Metal Complexes of Nano-Graphene Oxide to Thermal Decomposition of FOX-7

Chongmin Zhang [1], Xiaolong Fu [1,*], Xuexue Zhang [2], Jizhen Li [1], Xuezhong Fan [1] and Guofang Zhang [3]

[1] Xi'an Modern Chemistry Research Institute, Xi'an 710065, China; iceand010@163.com (C.Z.); fuxiaolong204@163.com (X.F.); jizhenli@126.com (J.L.); xuezhongfan@126.com (X.F.)
[2] Science and Technology on Combustion, Internal Flow and Thermostructure Laboratory, Northwestern Polytechnical University, Xi'an 710072, China; xuexuezhang@mail.nwpu.edu.cn
[3] Key Laboratory of Applied Surface and Colloid Chemistry, MOE/School of Chemistry and Chemical Engineering, Shaanxi Normal University, Xi'an 710062, China; gfzhang@snnu.edu.cn
* Correspondence: fuxiaolong204@163.com

1. Experimental part

Fourier-transform infrared (FTIR) spectra were tested on Bruker Tensor 27 using attenuated totalreflectance (ATR) method. Raman spectra were obtianed with a Renishaw inVia Raman spectrometer. The laser excitation was provided by a regular model laser operating at 514 nm. X-ray photoelectron spectroscopy (XPS) was tested on Thermo SCIENTIFIC K-Alpha. Scanning electron microscopy (SEM) and Energy Dispersive Spectrometer (EDS) images were taken with Hitachi SU8220. Differential scanning calorimeter (DSC) tests were performed on Netzsch DSC 200 F3 instrument with the heating rate of 10 ℃/min in nitrogen atmosphere (30 mL/min). TG-DSC-IR-MS coupling technology was performed on Netzsch STA449 F5, Bruker Vertex70, and Agilent Technologies 7890B GC, 5977A MSD with the heating rate of 10 ℃/min in helium atmosphere (50 mL/min).

1. Supporting Figures and Tables

Table S1 FTIR peaks and peak assignments for nGO, nGO-Cu and nGO-Fe.

Sample	FTIR absorption peak (cm^{-1})					
	**3000-3600**	**1700-1750**	**1500-1700**	**1150-1400**	**1000-1110**	**500-750**
nGO	3181	1730	1617	1374/1161	1039	-
nGO-Cu	3152	1729	1589	1390/1233	1051	608
nGO-Fe	3199	-	1602	1393	1068	694/590
Comments	Stretching of O-H	Stretching of C=O	Carboxylic and/or carbonyl moiety functional groups	Stretching of C-O in carboxy group and C-O-C stretching vibration for epoxy group	Stretching of C-OH bond	Stretching of Cu-O or Fe-O

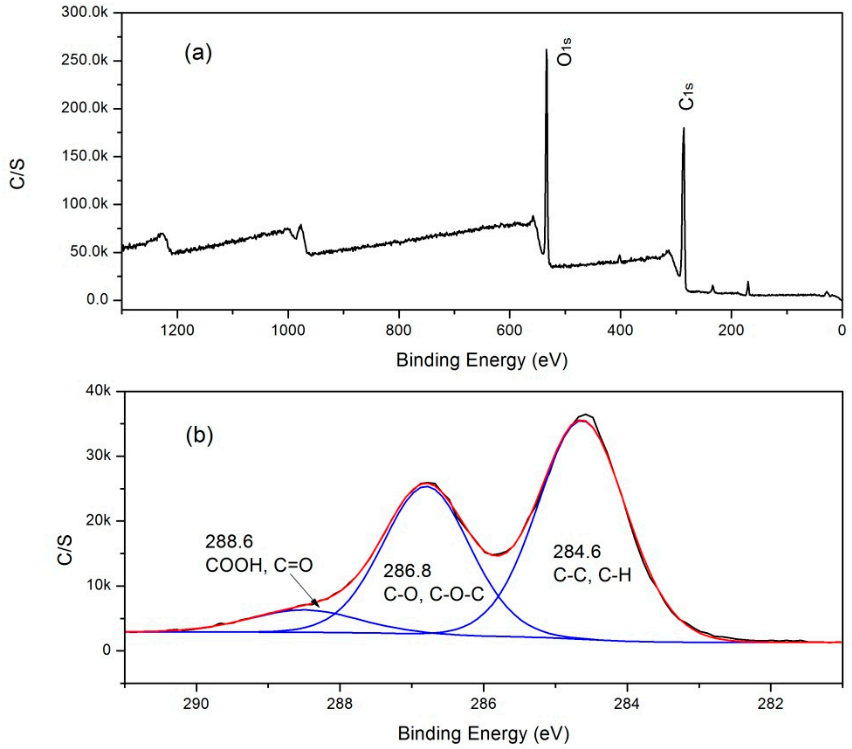

Figure S1 XPS binding energy spectra of (a) nGO and (b) the fitted C 1s peak curves for nGO.

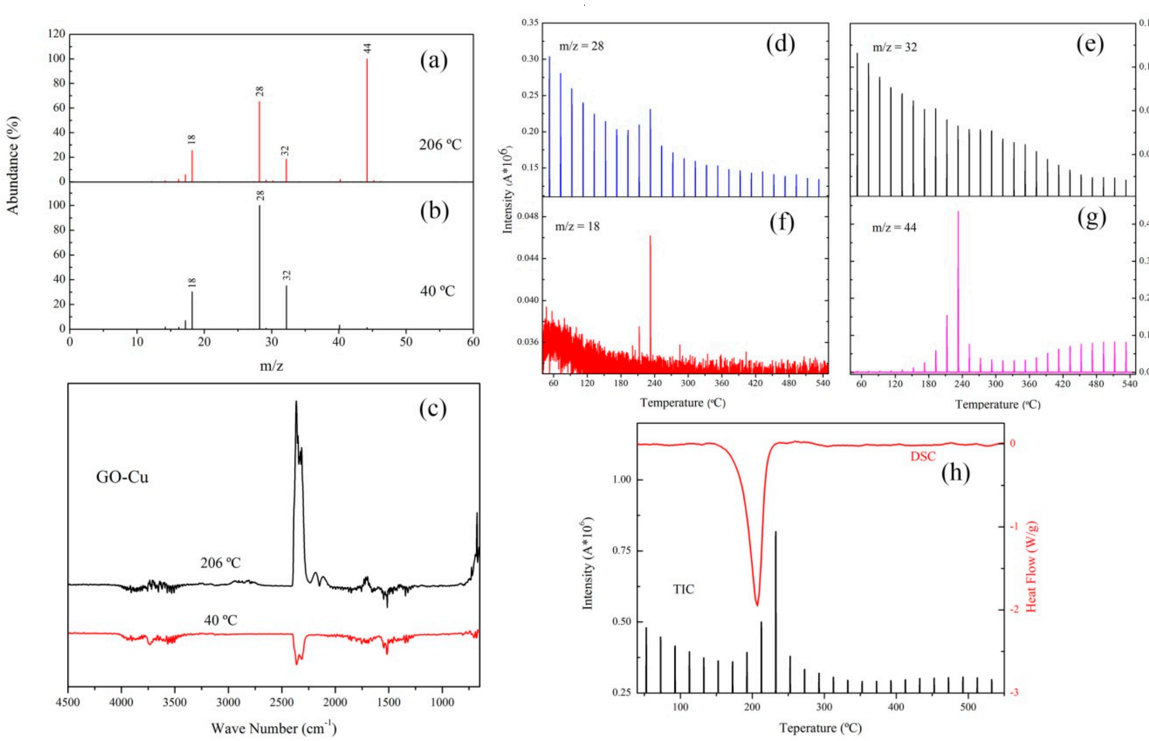

Figure S2. The MS spectra of nGO-Cu at (**a**) 206 °C and (b) 40 °C; (**c**) IR spectra of nGO-Cu at 206 °C and 40 °C, the ion current spectra of nGO-Cu in the decomposition with (d) m/z = 28, (**e**) m/z = 32, (**f**) m/z = 18, (**g**) m/z = 44, (h) total ion current.

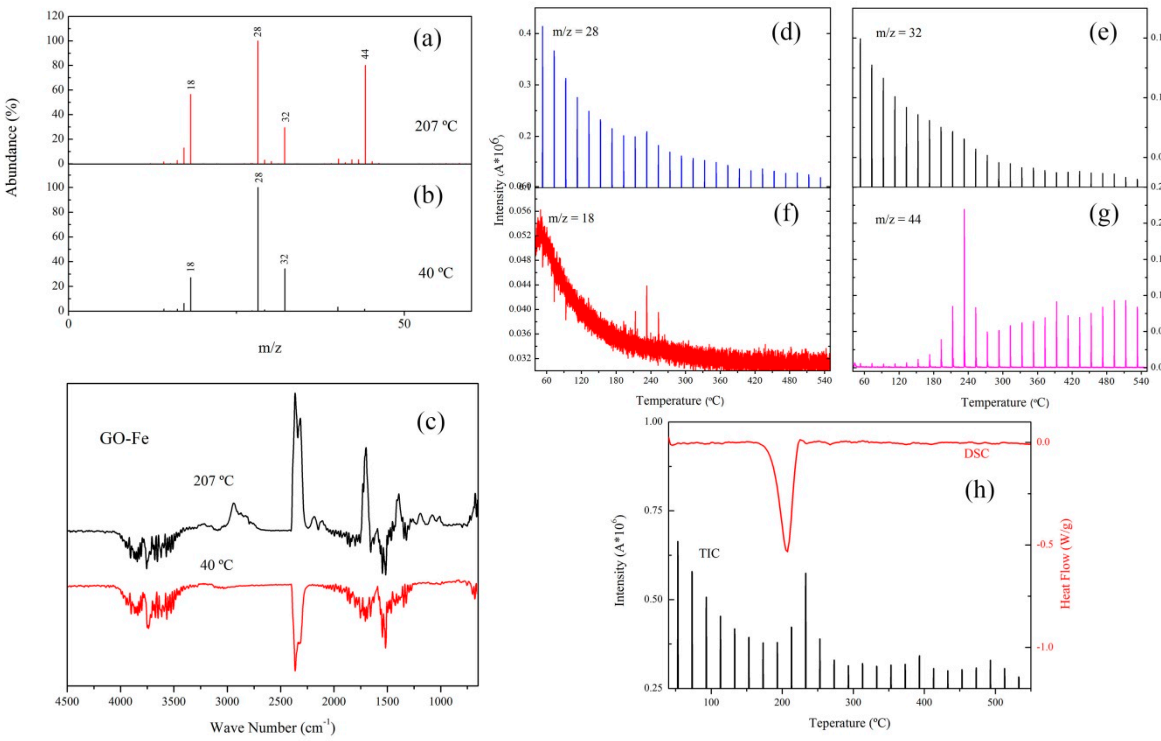

Figure S3. The MS spectra of nGO-Fe at (**a**) 207℃ and (**b**) 40℃; (**c**) IR spectra of nGO-Fe at 207℃ and 40℃; the ion current spectra of nGO-Fe in the decomposition with (**d**) m/z = 28, (**e**) m/z = 32, (**f**) m/z = 18, (**g**) m/z = 44, (**h**) total ion current.

Table S2. Raman shift of nGO **a**nd nGO-metal complexes.

sample	D-band peak		G-band peak		I_D/I_G
	shift	FWHM	shift	FWHM	
nGO	1353	108	1601	80	0.85
nGO-Cu	1353	186	1585	128	0.89
nGO-Fe	1353	175	1592	104	0.87

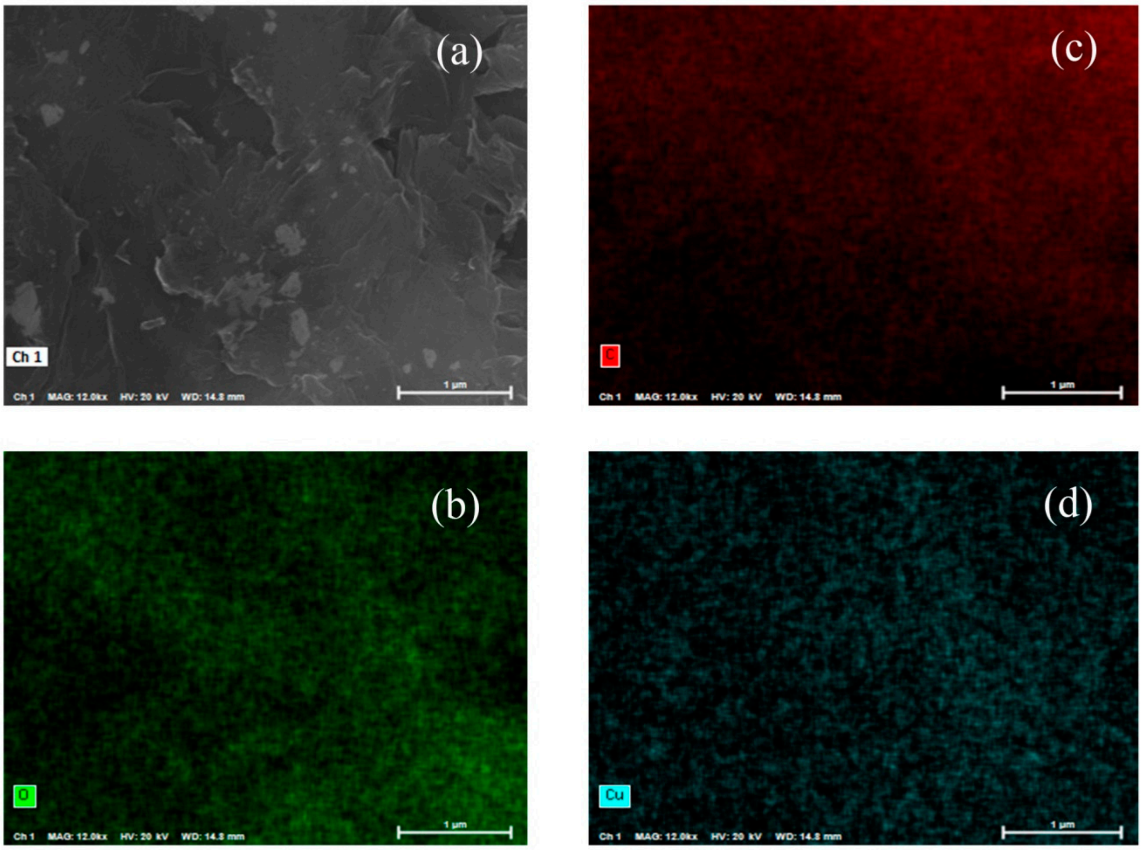

Figure S4 Mutil-elemental EDS mapping images of nGO-Cu: (**a**) SEM image, the distribution of **(b)** C, (c) O, (d) Cu atoms.

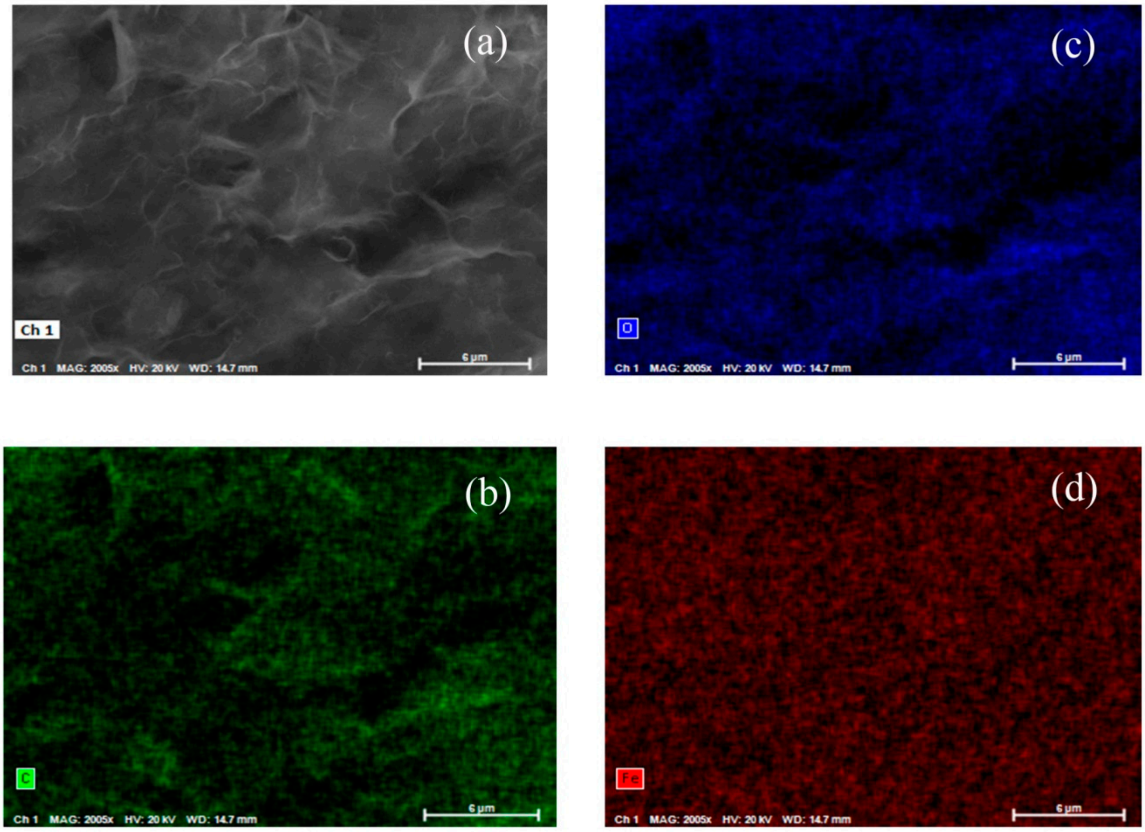

Figure S5 Mutil-elemental EDS mapping images of nGO-Fe: (**a**) SEM image, the distribution of **(b)** C, (c) O, (d) Fe atoms.

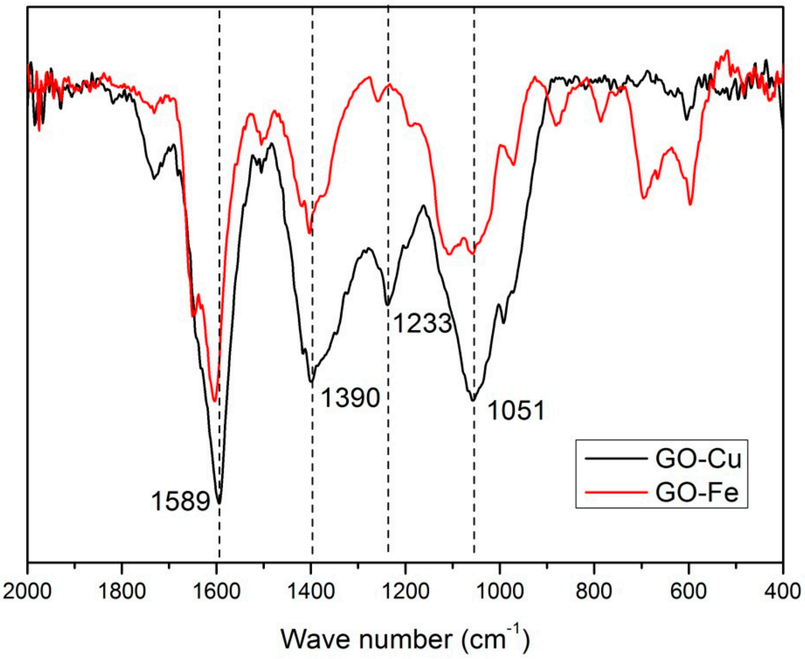

Figure S6 The comparison of GO-Cu and GO-Fe in FTIR.

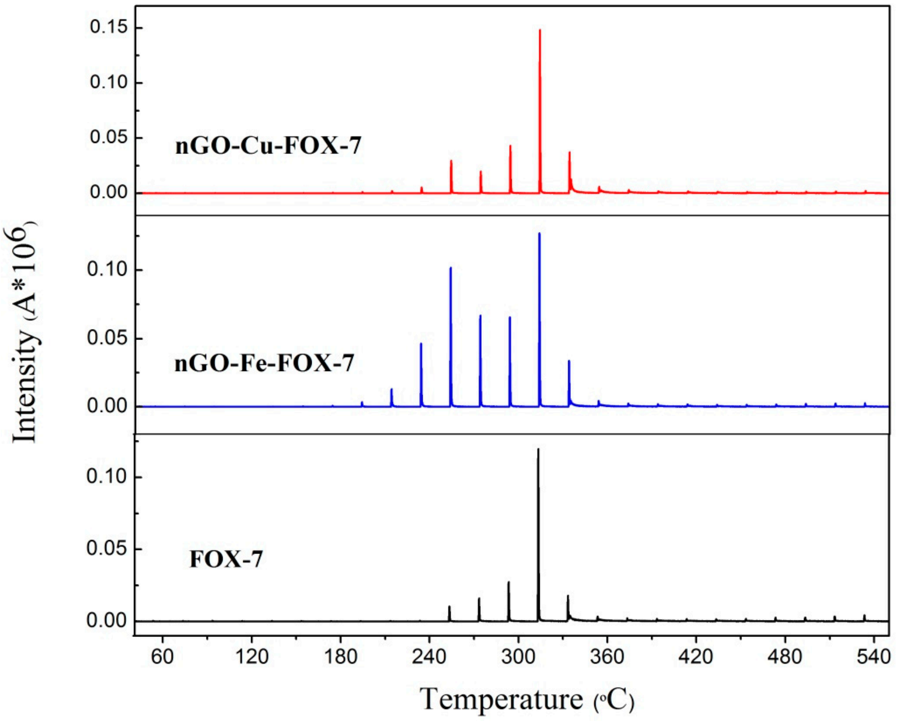

Figure S7 The ion current spectra of FOX-7, nGO-Fe-FOX-7 and nGO-Cu-FOX-7 in the decomposition with m/z = 30.
